# LVID-SLAM: A Lightweight Visual-Inertial SLAM for Dynamic Scenes Based on Semantic Information

**DOI:** 10.3390/s25134117

**Published:** 2025-07-01

**Authors:** Shuwen Wang, Qiming Hu, Xu Zhang, Wei Li, Ying Wang, Enhui Zheng

**Affiliations:** School of Mechanical and Electrical Engineering, China Jiliang University, Hangzhou 310018, China

**Keywords:** SLAM, dynamic environment, multi-sensor fusion, target detection, semantic mapping, geometric constraints

## Abstract

Simultaneous Localization and Mapping (SLAM) remains challenging in dynamic environments. Recent approaches combining deep learning with algorithms for dynamic scenes comprise two types: faster, less accurate object detection-based methods and highly accurate, computationally costly instance segmentation-based methods. In addition, maps lacking semantic information hinder robots from understanding their environment and performing complex tasks. This paper presents a lightweight visual-inertial SLAM system. The system is based on the classic ORB-SLAM3 framework, which starts a new thread for object detection and tightly couples the semantic information of object detection with geometric information to remove feature points from dynamic objects. In addition, Inertial Measurement Unit (IMU) data are employed to assist in feature point extraction, thereby compensating for visual pose tracking loss. Finally, a dense octree-based semantic map is constructed by fusing semantic information and visualized using ROS. LVID-SLAM demonstrates excellent pose accuracy and robustness in highly dynamic scenes on the public TUM dataset, with an average ATE reduction of more than 80% compared to ORB-SLAM3. The experimental results demonstrate that LVID-SLAM outperforms other methods in dynamic conditions, offering both real-time capability and robustness.

## 1. Introduction

Simultaneous Localization and Mapping (SLAM) technology, initially introduced in the late 1980s, has emerged as a fundamental core technology for robotic and autonomous navigation. In the last few years, many advanced SLAM systems have been developed, including MonoSLAM [1], PTAM [2], ORB-SLAM3 [3], DTAM [4], and DSO [5]. These classical algorithms have achieved good performance in specific environments. However, with fast progress in autonomous driving, drone exploration, and augmented reality, the application scenarios of SLAM technology have expanded from static structured environments to dynamic complex scenes, which places higher demands on the robustness and adaptability of algorithms. Currently, most classic algorithms still have low robustness in dynamic scenes, and when pedestrians or vehicles appear in the environment it leads to pose estimation failure. Traditional visual SLAM systems, such as ORB-SLAM2 [6], operate under the premise of a static scene and achieve real-time localization and mapping via sparse feature point matching. However, in dynamic scenes, dynamic object feature interference can easily cause trajectory drift or even system crashes. In recent years, inertial visual SLAM has effectively compensated for pose estimation errors caused by motion blur and rapid rotation by fusing IMU data with visual features. For example, ORB-SLAM3 demonstrates superior robustness in low-texture or lighting-varying scenes through a tightly coupled visual-inertial optimization model. However, since most SLAM systems cannot understand the surrounding environment through semantic information, their robustness in dynamic scenes remains low. To overcome this limitation, researchers have proposed combining traditional SLAM systems with deep learning techniques to improve robustness in dynamic situations. For example, DynaSLAM [7] integrates multi-view geometry with the Mask R-CNN [8] object detection network to significantly improve localization precision in highly dynamic environments by performing dynamic object segmentation and background restoration. DS-SLAM [9] achieves trajectory accuracy on the TUM dataset that is one order of magnitude higher than ORB-SLAM2 through the dual constraints of semantic segmentation networks and motion consistency checks. However, existing methods still have bottlenecks in real-time performance and computational efficiency, and the high time consumption of semantic segmentation limits the practical deployment of SLAM systems.

**Figure 1:**
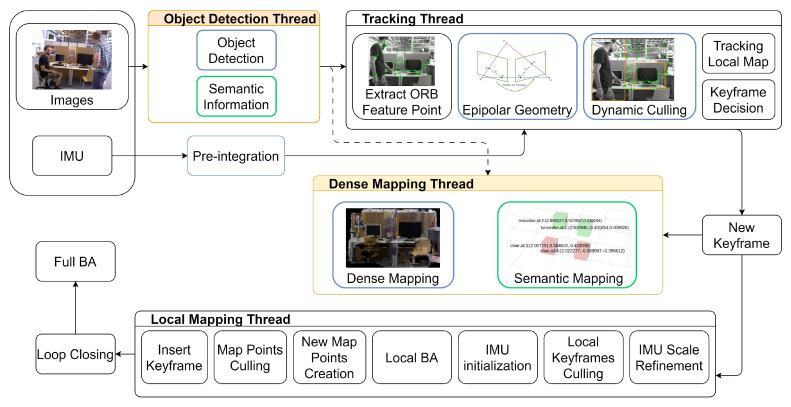
The overall framework of LVID-SLAM. LVID-SLAM performs object detection and semantic information acquisition through the object detection thread. The dense mapping thread uses keyframes to create dense octree maps and semantic maps.

An overview of the system is shown in Figure 1. This paper introduces a dynamic inertial-visual SLAM algorithm that combines geometric and semantic information to achieve motion estimation and real-time recognition of dynamic targets, thereby improving positioning precision and robustness while ensuring the system remains in real-time. The LVID-SLAM algorithm represents an advanced dynamic visual-inertial SLAM algorithm, developed upon the foundation of the ORB-SLAM3 system. LVID-SLAM deeply integrates object detection and geometric information and uses IMU information and visual sensors for tightly coupled pose estimation. The most significant of this paper’s contributions are listed below:

1. By integrating a lightweight Single Shot MultiBox Detector (SSD) [10] object detection algorithm with geometric methods, we propose a fast dynamic feature removal algorithm. While ensuring the fast recognition of dynamic objects, we combine geometric information to accurately preserve as many of the static feature points as possible, while eliminating dynamic objects and nearby feature points.
2. By integrating epipolar constraints and object detection techniques to reduce the number of feature points, the algorithm utilises IMU information to compensate for the deficiencies of tracking feature points in highly dynamic scenarios. Concurrently, IMU data are employed to address visual geometric line-motion degradation, thereby enhancing system robustness in highly dynamic scenarios.
3. A dense octree semantic map is established. Based on the classic ORB-SLAM3 sparse point cloud mapping, a dense point cloud map is established to provide richer texture information, and semantic information is imported to prepare for subsequent navigation and advanced tasks.

The rest of this paper is structured as below:

Section 2 discusses related work. Section 3 details our system methodology. Section 4 introduces the process and results of the experiments. Section 5 contains the conclusions.

## 2. Related Work

### 2.1. Based on Traditional Framework

For handling dynamic objects in SLAM systems, moving objects are typically treated as unwanted noise and swiftly removed by most methods. Zhang et al. [11] use optical flow residuals to identify dynamic semantic information from RGB-D point clouds and apply it to dynamic segmentation. They also use it for dynamic segmentation. Stückler and Behnke [12] segment RGB-D images into pixel regions. This is for motion estimation. However, this method is slow. Kim et al. [13] integrated an RGB-D camera with an IMU to estimate the camera’s pose. The IMU was employed to classify 3D feature points as either static or dynamic. An accurate rigid transformation matrix was calculated using the static feature points to eliminate moving objects. However, this method is limited by the use of an IMU and detection accuracy. Using RGB cameras to detect moving objects is also a related area of research [14].

Most techniques that rely on the use of geometric constraints employ the principles of epipolar geometry in order to distinguish between static and dynamic features. Constraints can be derived from multi-view geometry [15] or basic matrix estimation. It is worth noting that Tan et al. [16] proposed an online keyframe selection and update method to achieve adaptive dynamic environment modeling. The prior-based adaptive RANSAC [17] algorithm classifies points based on reprojection errors. Furthermore, the assumption of uniform texture distribution in static scenes is utilized to establish static points. However, this assumption may fail when the proportion of static objects is too small. Wang [18] proposed a method that is founded on both geometric constraints and mathematical modeling. By utilizing the disparity between consecutive frames, this method can effectively detect and identify moving objects in dynamic scenes. However, real-time processing in high-dynamic scenes remains a challenge.

### 2.2. Based on Deep Learning of Framework

The development of deep neural network technologies in recent decades has significantly improved robots’ semantic understanding of their surroundings. Against this backdrop, some research teams have started to incorporate image segmentation technology into visual SLAM systems to optimize their performance further.

DS-SLAM [9] uses a semantic segmentation neural network to achieve pixel-level segmentation and recognize moving targets. Combined with motion consistency checks, it effectively eliminates dynamic object interference, and the dense semantic octree map generated is easier to navigate. DynaSLAM [7], proposed by Bescos, combines dynamic object tracking and background restoration using ORB-SLAM2, enabling it to handle dynamic scenes in real environments, achieving higher accuracy than traditional visual SLAM methods in highly dynamic situations. Blitz-SLAM [19] innovatively eliminates noise blocks in local point clouds by integrating geometric and semantic information from the mask, depth images, and RGB images and generates a precise and clean global point cloud map through local point cloud generation. Additionally, SOF-SLAM [20], DRV-SLAM [21], and RDS-SLAM [22] are also image segmentation-based dynamic SLAM algorithms. However, these methods require pixel-level semantic segmentation, imposing high computational demands on the platform.

Detect-SLAM [23] uses object detection to identify static and dynamic objects in keyframes. It then uses motion probability matching to confirm the motion objects, combining this with the Random Sample Consistency (RANSAC) algorithm to eliminate outliers. This method deletes feature points on objects that are potentially moving, assigns an ID to each object, and builds a dense map of target objects. YOLO-SLAM [24] integrates the YOLOv3 [25] object detection model to recognise potential mobile objects in the scene. A geometric constraint method is also employed to get rid of dynamic features in the detected region. This method uses RANSAC-based depth differences to detect dynamic features, effectively removing feature points within the scope of dynamic objects. OVD-SLAM [26], Dynamic-VINS [27], Dynamic-SLAM [28], RLD-SLAM [29], and SG-SLAM [30] also adopt similar methods, combining depth images and depth thresholds to exclude background regions. Pan et al. [31] presented a dynamic feature segmentation SLAM algorithm using Mask R-CNN image segmentation. This algorithm utilizes optimized epipolar line geometry to remove dynamic outliers and enhance system robustness.

In recent studies, to improve the accuracy and efficacy of dynamic object recognition, some SLAM algorithms have incorporated tracking mechanisms for dynamic objects. The authors of Dynamic-SLAM [28,32] analyzed the benefits of estimating the motion status of dynamic objects in SLAM, using the relative displacement of static and dynamic feature points within a set threshold to track dynamic objects using qualified dynamic feature points. DynaSLAMII [33] and VDO-SLAM [34] utilize static elements for pose estimation and mapping, while also incorporating dynamic objects for pose estimation. They use the pose transformations of moving objects to assist navigation, which is advantageous for mapping in highly dynamic environments. However, since these methods employ optimization-based approaches to eliminate tracking and localization errors, they significantly increase computational complexity, thereby imposing a substantial additional computational burden on the system.

At present, mainstream SLAM technology systems still have significant gaps in real-time performance, which directly limits their development in dynamic scenarios. In practical application scenarios, composite factors such as dynamic environment interference, sensor data noise, and algorithm modeling deviations further increase the difficulty of accurate system modeling, resulting in insufficient adaptability and robustness. Especially in complex situations, existing solutions often have weak generalization capabilities. These technical bottlenecks pose multidimensional challenges to the industrial deployment of SLAM systems, and breakthrough innovations in algorithm architecture and engineering implementation are urgently needed.

## 3. Dynamic SLAM Methods and Strategies

This section will introduce the technical specifics of the LVID-SLAM system from six different angles. First, in the initial part, we present the system framework and the fundamental process. In the second part, we demonstrate the principle of the object detection algorithm. In the third part, we rigorously derive the geometric principles of the epipolar constraint approach. This approach is used to determine dynamic features. In the fourth part, we demonstrate the principle of removing dynamic objects. Then, in the fifth part, we propose how to use IMU to assist in position tracking. Finally, we propose a method for constructing a dense octree semantic map.

### 3.1. System Overview

When exploring autonomous robot navigation technology in dynamic environments, the reliability of pose estimation becomes a core metric for evaluating its performance. ORB-SLAM3 [3] is an advanced feature-based Simultaneous Localization and Mapping (SLAM) system that has demonstrated strong performance in a range of complex situations. It relies on efficient feature extraction and matching algorithms to estimate the robot’s pose accurately. The proposed LVID-SLAM system is built upon ORB-SLAM3 and incorporates three key parallel processes: tracking, local mapping, and loop closure detection. Numerous widely utilized public datasets have been employed to confirm the efficacy of ORB-SLAM3.

Therefore, LVID-SLAM chooses ORB-SLAM3 as the basic architecture to offer global localization and mapping functionalities. As Figure 1 shows, the LVID-SLAM system creates two additional parallel threads: a dense mapping thread and an object detection thread. These significantly enhance the efficiency of system operation through a multi-threaded coordination mechanism. Additionally, IMU pre-integration is realized based on a vision-inertial tight coupling architecture to compensate for the shortcomings of pure vision pose estimation and greatly enhance the system’s robustness.

First, semantic information can be obtained in the object detection thread to complete the dynamic attribute classification of the detected target while maintaining real-time performance. The input image is grayed out, ORB feature points are extracted, and multi-scale iterative calculations are performed using the Lucas–Kanade (LK) optical flow pyramid based on the extracted feature points to accurately analyze the motional pattern of feature points between frames. Combined with the dynamic region determined by object detection, the RANSAC algorithm is used to solve the fundamental matrix, significantly improving the precision of dynamic feature point removal. Since our system does not use time-consuming semantic segmentation methods, but instead uses a more efficient object detection algorithm, each thread can run cooperatively without blocking, ensuring the real-time operation of the system. Pure vision is affected by insufficient feature point extraction or visual degradation along epipolar lines, leading to miscalculations in position estimation. IMU sensors provide prior motion data, including velocity, rotation, and translation. For IMU pre-integration over a period of time, a relatively accurate position at the current moment can be estimated based on the position at the previous moment without being affected by light, compensating for the shortcomings of pure vision. In the dense mapping thread, a dense octree map is generated based on the sparse mapping output of ORB-SLAM3 [35]. The dense octree map not only contains richer texture information but also allows for more flexible real-time updates. Through the fusion of semantic information derived from the object detection thread, we generate a semantic map that supports advanced tasks. To reduce the system’s computational load, only keyframes are used for mapping during the local mapping process, effectively eliminating information redundancy between adjacent frames.

### 3.2. Object Detection Thread

**Figure 2:**
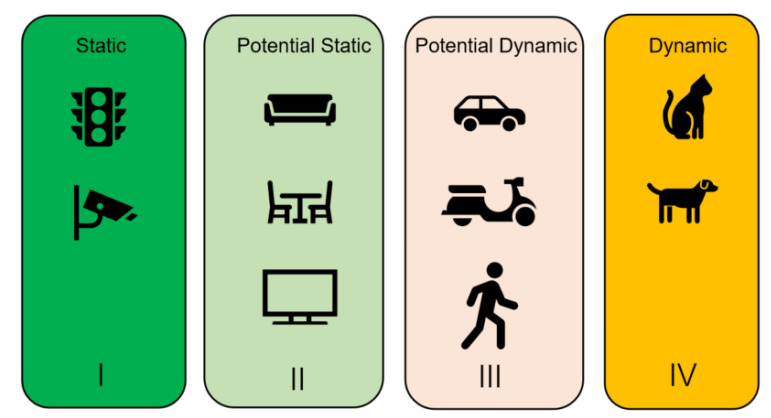
Dynamic object classification. Common objects are divided into four levels based on common knowledge.

A dynamic object detection thread is added in LVID-SLAM, with SSD [10] being adopted to perform object detection and obtain semantic information. Based on their experience and cognition, humans assign semantic information to objects in their surroundings. When memorizing unfamiliar environments, they naturally disregard dynamic objects, such as cars and people, while using prior knowledge to record stationary objects, such as trees and buildings. The SLAM system’s inability to interpret the semantic level of the surrounding environment will result in difficulties in the distinction between static and dynamic objects. SSD is used to locate dynamic objects within the input image in order to obtain their semantic labels. These semantic labels can effectively assist in completing object classification tasks. The identified targets are divided into different dynamic levels based on common sense. Level I (absolutely static targets) includes public facilities and backgrounds, which are generally assumed to be stationary; Level II (potentially static objects) has a certain probability of movement, including bottles, chairs, tables, sofas, dining tables, and televisions; Level III (potentially dynamic objects) has a high probability of movement, including vehicles and pedestrians; Level IV (dynamic objects) refers to freely moving individuals: mainly animals such as birds, dogs, cats, and sheep. Specific descriptions are shown in Figure 2.

These dynamic categories are assumed to be pre-trained and are typically present in everyday environments. If additional object categories are required, the model can be fine-tuned based on the PASCAL VOC 2007 [36] dataset. Object detection efficiency is crucial because maintaining real-time performance is critical for SLAM systems. Due to the lightweight SSD algorithm used, the object detection thread does not need to segment the edges of objects in detail like the semantic segmentation algorithm. Our system does not need to wait too long to cause system blockage. The lightweight SSD ensures the efficiency of object detection while ensuring the real-time performance of the system.

### 3.3. Epipolar Geometry

**Figure 3:**
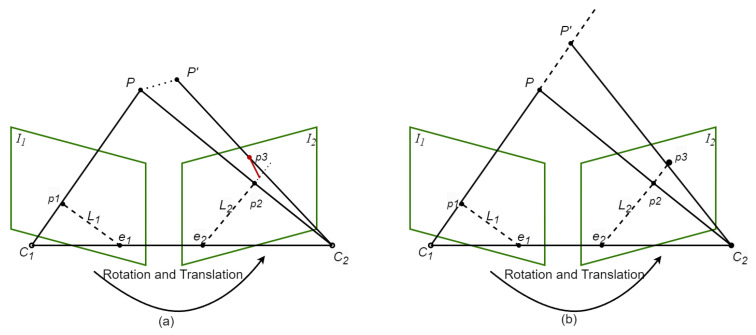
Epipolar geometry constraints for moving feature points. (**a**) Epipolar geometry constraints for dynamic feature points in general. (**b**) In special cases, dynamic feature points move along the polar line.

During object detection, potential dynamic objects—such as seated individuals or parked vehicles—are frequently identified. When solely employing deep learning-based object detection algorithms, feature points identified on the surfaces of humans and vehicles are classified as dynamic features. However, for people sitting still and vehicles with their engines turned off, their feature points still have value for pose estimation. Geometric methods are necessary to detect potential dynamic objects. In this part, epipolar geometry constraints are used to determine the dynamic points between adjacent frames. First, ORB feature points were acquired with the use of the Optical Flow Pyramid. Edge areas and dynamic feature points are removed, and the remaining stable feature points are used to generate a fundamental matrix with a high level of confidence. Using the epipolar geometry principle, the fundamental matrix calculation determines the deviation distances of feature points within the current frame from their corresponding epipolar lines within the two frames. The more different the deviation distance, the higher the possibility that the feature point belongs to an object that is moving. In multi-view geometry constraints, epipolar geometry is used to analyze whether feature points tend to be static or dynamic. In general, only static elements satisfy the epipolar constraint. As shown in Figure 3:

- *P*: The map point detected in adjacent frames $I_{1}$ and $I_{2}$ by the camera at the same position;
- $P^{\prime }$: The map point where map point *P* is moved;
- $p_{1}$: This image shows map point *P* within frame $I_{1}$;
- $p_{2}$: This image shows map point *P* within frame $I_{2}$;
- $C_{1}$ and $C_{2}$: These are the optical centres of frames $I_{1}$ and $I_{2}$;
- Baseline: The line connecting the optical centers $C_{1}$ and $C_{2}$;
- Epipole ($e_{1},e_{2}$): The point at which the baseline intersects with the image planes $I_{1}$ and $I_{2}$, respectively;
- Epipolar Plane: The plane is determined by the baseline and the map point *P*;
- Epipolar Lines ($L_{1},L_{2}$): The lines are formed by the epipolar plane intersecting the image planes $I_{1}$ and $I_{2}$;
- $p_{3}$: The lines are formed by the epipolar plane intersecting the image planes $I_{1}$ and $I_{2}$.

The $p_{1},p_{2}$, and $p_{3}$ homogeneous coordinates are defined as $P_{1},P_{2}$, and $P_{3}$, as follows:

(1) $$P_{1}=\left[x_{1},y_{1},1\right],\;P_{2}=\left[x_{2},y_{2},1\right],\;P_{3}=\left[x_{3},y_{3},1\right],$$

The image pixel coordinate system is used to represent the coordinate values of the feature points as *x* and *y*.

The following formula can be used to calculate the epipolar line $L_{2}$ of the current frame, with the fundamental matrix $F_{M}$ being calculated from the optical flow pyramid:

(2) $$\begin{array}{c} L_{2}=\left[\begin{array}{c} X\\ Y\\ Z \end{array}\right]=F_{M}P_{1}=F_{M}\left[\begin{array}{c} x_{1}\\ y_{1}\\ 1 \end{array}\right]. \end{array}$$

Next, the offset distance *D* is defined as the distance between the feature point $P^{\prime }$ and its corresponding epipolar line. The expression of the offset distance is as below:

(3) $$D=\frac{\left|P^{\prime }F_{M}P\right|}{\sqrt{X^{2}+Y^{2}}}.$$

If the feature point *P* is stationary, the offset distance should be zero. However, due to noise and other uncertainties, the offset distance is usually non-zero, though it remains under the empirically determined threshold, denoted by the symbol ξ. As shown in Table 1, for the empirical threshold ξ, usually if the threshold ξ is too large, when the ξ is greater than 0.6 the system cannot recognize too many dynamic feature points, resulting in a large pose error, affecting the overall pose estimation of the system. If the ξ is set too small, some static feature points will be regarded as dynamic feature points and removed, resulting in too few feature points, and a large number of available static feature points will be mistakenly removed, which will affect the subsequent local mapping. Therefore, we finally chose a threshold of 0.4, which can not only not affect the overall pose estimation but also ensure that there are enough available feature points. When point *P* moves to point $P^{\prime }$, the calculated value of *D* is usually larger compared to the empirical threshold ξ. As shown in Figure 3a, the red line denotes the offset distance *D*. It is impossible to determine whether or not a feature point is moving based on visual reprojection error when it moves in the direction of the epipolar line. If, in Figure 3b, feature point *P* moves along the extension line of $C_{1}P$, when point *P* moves to $P^{\prime }$, the projection point on the polar plane is $p_{3}$, and $p_{3}$ is still on epipolar line $L_{2}$. Calculating *D* using Formula (3) gives a value of zero, but in this special case the visual reprojection error actually degrades and fails.

### 3.4. Dynamic Object Removal Strategy

In reality, all objects can be categorized as either dynamic or static. If only object detection is performed, for a person sitting quietly on a chair, object detection will, based on prior information, most likely regard it as a dynamic object and filter out feature points that can be used for local mapping. Or, if a person is walking and holding a book, the book is in dynamic motion at that moment. If such potential dynamic objects are not correctly filtered out, it will affect the subsequent pose estimation. Our approach is to first filter out dynamic objects and potential dynamic objects using dynamic prior information by using the object detection thread. As shown in Figure 2, different weight values are assigned to different objects. The probability of being judged as dynamic increases with the size of the value.

The Optical Flow Pyramid is used to establish a relatively reliable fundamental matrix, which is then employed to detect if feature points are moving or stationary. If the dynamic feature points of the epipolar geometry test overlap with the results of the object detection as dynamic objects, they can be judged as dynamic. A relatively reliable fundamental matrix can be employed to extract dynamic feature points from potential dynamic objects by applying limit constraints.

The specific dynamic feature point Culling algorithm is shown in Algorithm 1.
**Algorithm 1** Dynamic Object Culling Algorithm**Input:** 
Previous frame’s feature points, $P_{p}$; Current frame’s feature points, $P_{c}$; Previous frame’s static feature points, $P_{psta}$; Current frame’s static feature points, $P_{csta}$; Previous frame, $F_{p}$; Current frame, $F_{c}$; Dynamic object culling threshold, ξ; Number of feature points threshold, $N_{th}$;**Output:** 
The set of dynamic feature points in the current frames, $S_{t}$;1:$P_{c}=CalcOpticalFlowPyrLK\left(F_{p},F_{c}\right)$;2:Culling dynamic feature points at the edges;3:**for** each matched pair $P_{psta},P_{csta}$ in $P_{p},P_{c}$ **do**4:   **if** $!(InDynamicRegion\left(P_{p}\right)$) **then**5:       Append $P_{p},P_{c}$ to $P_{psta},P_{csta}$6:   **end if**7:   Feature++8:**end for**9:**if **$Feature>N_{th\;}$** then**10:  $Fun\_Mat=FindFundamentalMat(P_{psta},P_{csta},RANSAC)$; 11:**else if **$Feature\leq N_{th}\;\mathrm{and}\;DynamicObjectsFlag$  **then**12:   $\;Fun\_Mat=FindFundamentalMat(P_{p},P_{c},RANSAC)$;13:**end if**14:**for** each matched pair $p_{p},p_{c}$ in $P_{p},P_{c}$ **do**15:    **if** ($\mathrm{CalcEpiLineDistance}(p_{c},p_{p},Fun\_Mat)<\xi$) **then**16:       Append $p_{c}$ to $S_{t}$17:    **end if**18:**end for**

### 3.5. Position Estimation Using IMU Pre-Integration

There are still significant limitations when using only a camera as a sensor. As a low-frequency observation device, cameras can provide rich visual information, but the quality of their data is limited by external lighting conditions and highly dynamic scenes. The effectiveness of visual information is significantly reduced, greatly reducing the robustness of SLAM systems. Additionally, pure vision may also suffer from the phenomenon of feature point degradation along polar lines. In contrast, IMU observations have a higher frequency, which can partially bridge the gaps between discrete visual observation frames and enhance the direct correlation between frames. As a sensor sensitive to internal states, IMU observations generally do not change significantly due to the appearance of external moving objects, even when visual reprojection degrades, providing a good complement to visual sensors. However, IMUs also have their own issues, such as long-term drift and error accumulation. These issues prevent IMU from being used alone for long-term pose estimation, necessitating the continuous calibration of IMU parameters and estimation results using visual data.

The following equation shows that, in frame i, when there are sufficient feature points, the visual odometer can function normally, and the camera coordinates $P_{c}^{i}$ can be obtained through the world coordinate system $P_{w}^{i}$ and the transformation matrix $T_{cw}^{i}$ to obtain a relatively accurate $P_{c}^{i}$. The specific formula is as follows:

(4) $$P_{c}^{i}=T_{cw}^{i}\cdot P_{w}^{i},$$

If there are too many dynamic objects or insufficient light, causing the camera to collect too few feature points, it is impossible to determine $T_{cw}^{i}$ in the above equation, and using only the visual sensor may cause the pose estimate for the current frame to fail. If visual loss tracking is lost, the pre-integration of the IMU can be used to calculate the camera’s current position.

**Figure 4:**
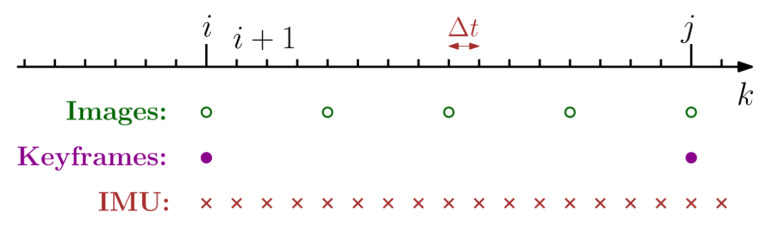
Different rates of the camera and IMU. Keyframes are selected from normal images based on appropriate conditions, and the IMU is a high-frequency sensor relative to the camera, so the frequency of the IMU is higher than that of normal images.

As shown in Figure 4, between two successive keyframes k = i and k = j, the iteration interval of the IMU between the two keyframes is Δt, which is shorter than the iteration time of the keyframes. The IMU data are relatively reliable in the short term. Due to the short IMU iteration interval, we assume that the error in the pre-integration process can be ignored. The definitions of short-term rotation, velocity, and position increments are from [37], as shown below:

(5) $$\begin{array}{cc} \Delta \;{\tilde{R}}_{\mathrm{ij}} & =R_{i}^{T}R_{j}, \end{array}$$

(6) $$\begin{array}{cc} \Delta {\tilde{v}}_{\mathrm{ij}} & =R_{i}^{T}\left(v_{j}-v_{i}-g\Delta t_{\mathrm{ij}}\right), \end{array}$$

(7) $$\begin{array}{cc} \Delta {\tilde{p}}_{\mathrm{ij}} & =R_{i}^{T}\left(p_{j}-p_{i}-v_{i}\Delta t_{\mathrm{ij}}-\frac{1}{2}\;g\Delta t_{\mathrm{ij}}^{2}\right). \end{array}$$

Visual inertial tight coupling optimization can be regarded as a minimum optimization problem for keyframes: each frame contains rotation, translation, velocity, gyroscope, and accelerometer, and a set of k + 1 frame state values is given as follows:

(8) $${\bar{Y}}_{i}≐\left\{Y_{0},\ldots ,Y_{k}\right\},$$

A set of *l* three-dimensional point state values is also given:

(9) $$X≐\{x_{0},\ldots ,x_{l-1}\},$$

The final error (reprojection error and inertial residual) is expressed by the following equation, which is given in reference [3]:

(10) $$\min_{{\bar{Y}}_{k},X}\left(\sum_{j=0}^{l-1}\sum_{i\in K^{j}}\rho_{H}\left({∥r_{ij}∥}_{\sum_{ij}^{-1}}\right)+\sum_{i=1}^{k}{∥r_{I_{i-1,i}}∥}_{\sum_{I_{i,i+1}}^{-1}}^{2}\right).$$

where $r_{ij}$ denotes the visual reprojection error and $\rho_{H}$ denotes the robust kernel function, which serves to regulate the visual reprojection error, where $r_{I_{i-1,i}}$ denotes the inertial residual, as expressed in [3] as follows:

(11) $$r_{I_{i,i+1}}=\left[r_{\Delta R_{i,i+1}},r_{\Delta v_{i,i+1}},r_{\Delta p_{i,i+1}}\right],$$

(12) $$r_{\Delta R_{i,i+1}}=\mathrm{Log}\left(\Delta R_{i,i+1}^{T}R_{i}^{T}R_{i+1}\right),$$

(13) $$r_{\Delta v_{i,i+1}}=R_{i}^{T}\left(v_{i+1}-v_{i}-g\Delta t_{i,i+1}\right)-\Delta v_{i,i+1},$$

(14) $$r_{\Delta p_{i,i+1}}=R_{i}^{T}\left(p_{j}-p_{i}-v_{i}\Delta t_{i,i+1}-\frac{1}{2}g\Delta t^{2}\right)-\Delta p_{i,i+1}.$$

$K^{j}$ is a set of keyframes of the observed 3D points. If the visual reprojection error is large, the overall error will also be large, affecting the system’s overall accuracy. Through the adjustment of the kernel function $\rho_{H}$, this error can be reduced when the visual error is large.

### 3.6. Dense Octree Semantic Mapping

ORB-SLAM3 itself has the function of constructing sparse point cloud maps. Although computationally efficient, the resulting sparse point clouds lack detailed environmental information and are unsuitable for applications requiring high-precision surface reconstruction or dense mapping. In addition, the original mapping function has limited robustness in dynamic environments. LVID-SLAM employs dense octree maps for semantic mapping while retaining the sparse point cloud maps provided by ORB-SLAM3. Octree mapping divides three-dimensional space into progressively smaller voxels through recursive division. Unoccupied empty areas can be skipped directly, saving storage and computing resources.

The octree only stores information about occupied or regions of interest, while unoccupied space is represented at a lower resolution, avoiding wasted storage space. The octree allows for real-time dynamic updates to the map, making it useful for robotic mapping and exploration in dynamic scenes. Octree nodes can be merged when sensor data are updated, and the octree can dynamically insert, delete, or update point cloud data to adapt to dynamically changing environments.

The octree map contains semantic information passed by the object detection thread. Semantic information is associated with the bounding box regions. In LVID-SLAM, dense mapping does not use every frame of the image but instead maps based on keyframes generated by the tracking thread, significantly improving mapping efficiency. First, ordered 3D points are generated based on the transformation matrix of the keyframes and depth image. Depth values that are not within the preset threshold range are discarded, and the remaining point cloud generates a local point cloud map. Next, the octree map is integrated with semantic data from the object recognition thread. To remove dynamic object point clouds, the current pixel is determined to be inside or out of the dynamic object detection box, and only pixels outside the detection box are used for mapping. Then, the local point cloud is converted and updated to the octree map. The map is voxel-filtered to optimize noise and reduce point cloud density. Statistical filtering is used to calculate the standard deviation and average value of the neighborhood of points in the point cloud. To optimize point cloud quality, outlier points exceeding the threshold are removed. After constructing the octree map, a semantic mapping is created with semantic information, and objects detected by object detection are displayed by cubes of different colors. The center of gravity coordinates of different semantic objects are obtained through a 3D point cloud, and finally, the processed octree map and semantic map are converted to ROS format for publication.

## 4. Experimental Results

In order to evaluate the operational capability of our algorithm in dynamic environments, we primarily used the below publicly accessible datasets to verify our system, including the TUM dataset [38] and the KITTI dataset [39]. These datasets provide dynamic scenes for testing the capabilities of detection algorithms. The experiments primarily used two types of errors to assess the localization accuracy of each framework. Absolute Trajectory Error (ATE) and Relative Pose Error (RPE) are used, where ATE is only compared to the translation part and RPE contains the relative translation error and the relative rotation error. Given the adequacy of the data, we primarily use Root Mean Square Error (RMSE), Mean, Median, and Standard Deviation (Std.) to describe the results, and Improvements to describe the rate of improvement. Then, ablation experiments are conducted to assess the impact of different algorithms on system accuracy. Next, the dynamic filtering effect and mapping effect are demonstrated. Finally, the system’s runtime is compared and evaluated. Considering the random volatility of the experimental results, for the following sequences under each dataset, twenty experiments were performed, the maximum and minimum values were removed, and then the average value of other data was taken. All algorithms were mainly experimented on a computer equipped with a Ubuntu 18.04 operating system, AMD Ryzen 7 7845H CPU, NVIDIA GeForce GTX 4060 GPU, and 16 GB memory.

### 4.1. Evaluation on the TUMRGB-D Dataset

The TUM RGB-D dataset is widely used in academic research. This dataset acquires RGB-D images and covers various indoor scenes. To demonstrate the capabilities of the LVID-SLAM system in dynamic situations, five sequences were chosen from the dataset, all of which belonged to the dynamic object category. The first four sequences are scenes with a high dynamic level, and the fifth sequence is a supplementary scene with a low dynamic level. The specific dataset includes fr3_sitting_static, fr3_walking_xyz, fr3_walking_static, fr3_walking_rpy, and fr3_walking_half. In these sequences, ‘walking’ and ‘sitting’ represent dynamic poses of human objects, with ‘sitting’ indicating low-dynamic scenes and ‘walking’ representing high-dynamic scenes. Additionally, ‘xyz’, ‘static’, ‘rpy’, and ‘half’ correspond to different camera poses.

To assess the improvement in its performance, the LVID-SLAM system is evaluated against the original system using the experimental results of ORB-SLAM3. We present the results of the evaluation comparisons for the five dynamic scenario sequences in Table 1, Table 2 and Table 3. Additionally, we provide the trajectory improvement rate of LVID-SLAM relative to the original ORB-SLAM3 localization. The improvement rate calculation formula in the tables is as follows:

(15) $$\eta =\frac{o-r}{o}\times 100\%,$$

where *o* represents the absolute trajectory error of ORB-SLAM3, *r* represents the absolute trajectory error of LVID-SLAM, and η represents the rate of improvement of the trajectory of LVID-SLAM with respect to ORB-SLAM3.

This experiment is based on ORB-SLAM3 with modifications. In order to evaluate the improvement in performance of LVID-SLAM, a comparison with ORB-SLAM3 is shown in Table 1, Table 2 and Table 3. Table 1 provides the ATE metric, which evaluates translation in meters per second. Table 2 and Table 3 show the RPE metric evaluated by our system, considering both translation and rotation, with translation in meters per second and rotation in degrees per second.

As shown in Table 2, Table 3 and Table 4, SLAM does not show a significant improvement over ORB-SLAM3 in the low dynamic fr3_sitting_static. However, in high-dynamic datasets, LVID-SLAM demonstrates substantial improvements over ORB-SLAM3 in most metrics. Therefore, the proposed LVID-SLAM achieves significantly better pose estimation performance than ORB-SLAM3 in environments with high-dynamic objects. In high-dynamic datasets our algorithm is more than 80% better than ORB-SLAM3, and in low-dynamic datasets our algorithm is more than 10% better than ORB-SLAM3.

**Figure 5:**
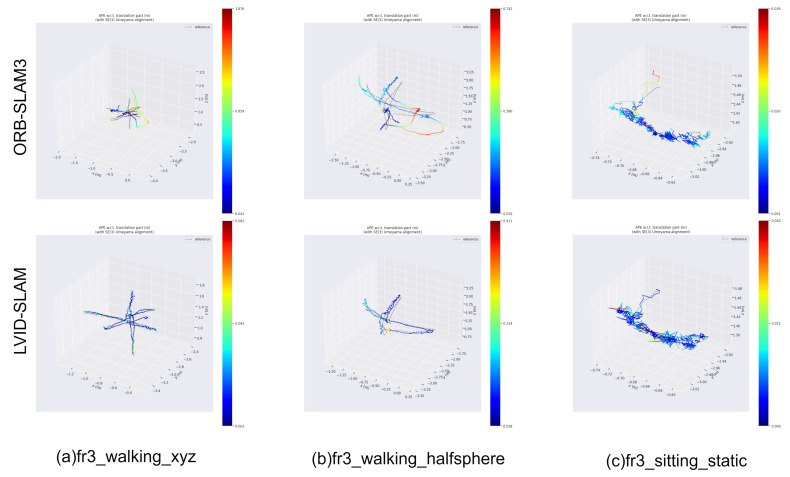
The TUM RGB-D dataset was used to generate trajectory results for ORB-SLAM3 and LVID-SLAM.

To visually observe the pose estimation output of our system, as shown in Figure 5, LVID-SLAM achieves significantly higher pose estimation accuracy than ORB-SLAM3 in high-dynamic scenes across three different datasets. As shown in Figure 5, red indicates a higher pose estimation error along the trajectory, while blue indicates a lower error. On datasets a–b, LVID-SLAM achieves much higher pose estimation accuracy than ORB-SLAM3. The overlap rate between the true trajectories of ORB-SLAM3 and estimated trajectories is low, with lower overlap rates corresponding to redder estimated trajectories. For the low-dynamic scene sequence c, the performance of LVID-SLAM compares favorably with that of ORB-SLAM3.

**Figure 6:**
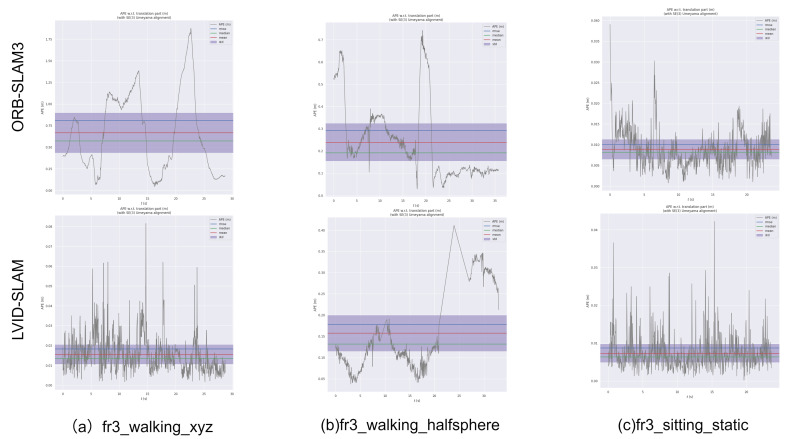
The TUM RGB-D dataset was used to generate trajectory results for ORB-SLAM3 and LVID-SLAM.

As shown in Figure 6, the LVID-SLAM pose estimation error is one magnitude lower than that of ORB-SLAM3 in datasets a–b. ORB-SLAM3 exhibits larger ATE fluctuations, while LVID-SLAM demonstrates smaller ATE fluctuations. This suggests that LVID-SLAM exhibits enhanced robustness in dynamic scenes. For the low-dynamic scene sequence c, however, LVID-SLAM shows a smaller improvement over ORB-SLAM3.

In order to further analyze the effectiveness of the proposed algorithm, we will continue to compare it with similar algorithms, such as ORB-SLAM3, DS-SLAM, and RDS-SLAM. The results of these comparisons are presented in Table 5. ORB-SLAM3 exhibits poor localization precision in dynamic environments. DS-SLAM uses semantic segmentation for dynamic target recognition, so it outperforms our system in some sequences. However, from the system’s real-time performance in Table 4, DS-SLAM is far inferior to our system. Overall, the experimental results conclude that LVID-SLAM achieves state-of-the-art performance.

### 4.2. Evaluation on the KITTI Dataset

**Figure 7:**
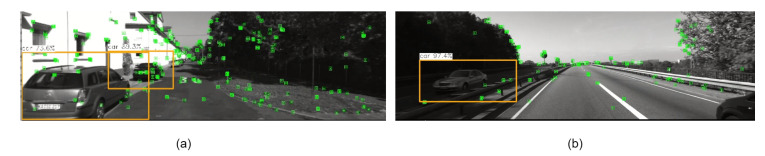
Valuation of KITTI tracking dataset. (**a**) is a dynamic object such as a vehicle that appears multiple times in the dataset. (**b**) is a highway scene that appears in the dataset.

The LVID-SLAM system demonstrates excellent performance when using RGB-D as input and maintains excellent performance when using other types of input. We used the KITTI dataset for experiments. The KITTI dataset is primarily an outdoor stereo dataset. Due to hardware limitations on RGB-D depth, acquiring long-range RGB-D data is a significant challenge, so stereo cameras are typically used to record the dataset. The KITTI dataset consists of 21 sequences, with the first 11 sequences providing official ground truth trajectories for direct trajectory comparison. Therefore, these first 11 sequences were used for experiments. The 00–10 sequences used in the experiment mainly describe urban or rural streets and roads. The types of sensors are also very rich, including visual, lidar, inertial odometers, and GPS. In this experiment, fusion data from visual and IMU sensors are required. According to the KITTI dataset official website, the odometry dataset only contains 10 Hz visual information. It is necessary to download 100 Hz IMU data from the raw data dataset and use a Python script to align the timestamps of the two. We compare LVID-SLAM with the original ORB-SLAM3 algorithm. In most sequences, LVID-SLAM shows a certain improvement in performance. As shown in Table 6, for the 11 sequence datasets 00–10, sequences 00, 02, 03, and 09 have a high number of dynamic objects (see Figure 7a), which results in significant pose estimation errors. For the 01 dataset, the vehicles primarily travel at high speeds, as shown in Figure 7b, with faster speeds and a higher density of vehicles, resulting in larger overall pose estimation errors. However, the performance is still significantly improved compared to ORB-SLAM3. The 08 sequence involves frequent transitions between sunlight and shade, along with moving objects of various sizes, such as vehicles and pedestrians. These factors have a significant impact on visual localization. For the 04 and 07 sequences, there are few dynamic objects, mainly static vehicles, and the overall pose estimation error is small. We can see that, in the 05 sequence, ORB-SLAM3 is better than our system. Due to the intersections and multiple narrow sharp turns in the 05 sequence, the instantaneous uncertainty of dynamic object detection increases, resulting in some valuable static background feature points in the key frame being mistakenly removed. Our system has a larger pose estimation error in the 05 sequence.

### 4.3. Ablation Experiment

LVID-SLAM can be regarded as an enhanced version of ORB-SLAM3. We added object detection and epipolar geometry to ORB-SLAM3 to verify its effectiveness. From the third column of Table 7, it can be seen that the epipolar geometry module enhances the system’s positioning precision in dynamic environments to a certain extent. From the fourth column, using the object detection thread alone, the pose error has a higher improvement than using only the geometric method, but because pure object detection will remove all feature points inside the object detection box, a large number of static feature points available inside the object detection box will be wasted. From the fifth column, we can see that, after combining object detection and epipolar geometry, all indicators of absolute trajectory error are below 0.02, greatly boosting the system’s positioning precision in dynamic scenarios.

**Figure 8:**
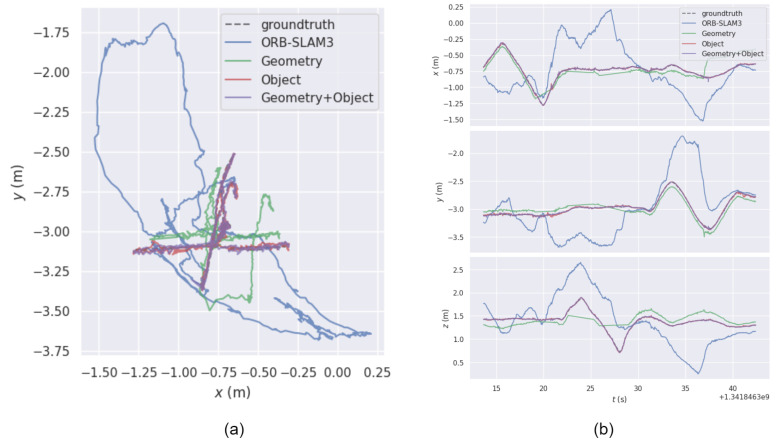
Trajectory results from ablation experiments using the TUM RGB-D dataset. (**a**) The trajectory projection in the xy plane. (**b**) The trajectory error in the x, y, and z axes.

In Figure 8, Figure 8a shows the trajectory projection in the xy plane, and Figure 8b shows the trajectory error in the x, y, and z axes. The blue line depicts the trajectory estimated from the ORB-SLAM3, and the dotted line represents the true reference trajectory. It can be clearly seen that there is a large gap between the two trajectories, and the trajectory drifts significantly. The green trajectory intuitively shows that, when using only epipolar geometry, there is a certain offset in the pose estimation error between the estimated and true trajectories. However, this is greatly improved compared to ORB-SLAM3. The red line represents the curve drawn using the object detection algorithm alone. The purple trace represents the combination of object detection and epipolar geometry. Since the pose error of adding geometry to object detection and using object detection alone is small, most of the trajectories of the red and purple curves are close to the true trajectory. As can be seen, combining object detection with epipolar geometry improves the system’s robustness in environments with high motion.

### 4.4. Dynamic Removal Effect and Mapping Effect

**Figure 9:**
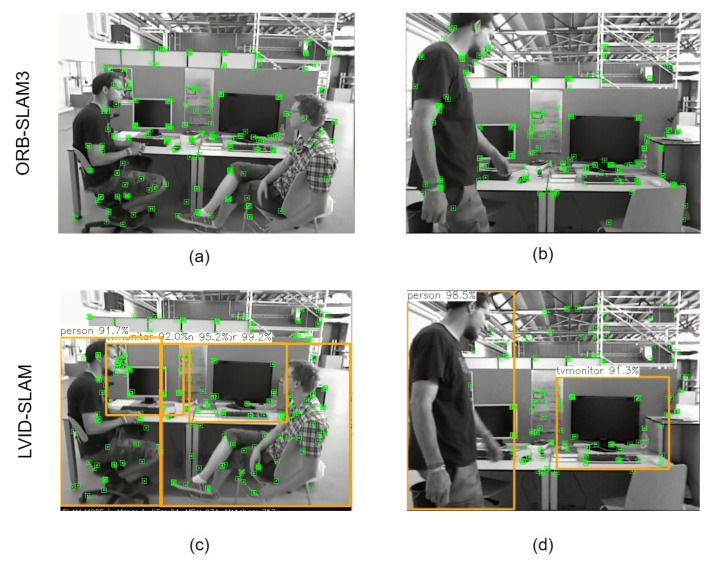
Comparison of dynamic feature point culling effect of ORB-SLAM3 and LVID-SLAM. (**a**) ORB-SLAM3 in low dynamic scenes. (**b**) ORB-SLAM3 in a high-dynamic scene. (**c**) LVID-SLAM in low dynamic scenes. (**d**) LVID-SLAM in high-dynamic scenes.

To illustrate the effectiveness of the motion region for potential motion detection in LVID-SLAM, the following comparison experiments were conducted. Figure 9a,b illustrate the results of ORB-SLAM3, while those of LVID-SLAM are shown in Figure 9c,d. ORB-SLAM3 and LVID-SLAM were run on two different scenes: Figure 9a,c show scenes with stationary people, while Figure 9b,d show scenes with walking people. In the seated scene, the movement amplitude of the people is small, making them potential dynamic objects; in the walking scene, the movement amplitude of the person is large, making them typical dynamic objects. In Figure 9a, ORB-SLAM3 correctly annotated the feature points, which are mainly concentrated on the static background. The seated people, due to their small movement amplitude, retain their feature points. This indicates that ORB-SLAM3 has high robustness when handling static backgrounds. However, in Figure 9b, ORB-SLAM3 still retained a high proportion of feature points in scenes containing dynamic objects (e.g., walking people). In Figure 9c, LVID-SLAM identifies sitting people as potential dynamic targets. People occupy a large portion of the image. They have small ranges of motion. Through the epipolar geometry method, they are judged to be static objects, and their feature points are not filtered out in large numbers, retaining the key feature points of potential dynamic objects. In Figure 9d, LVID-SLAM effectively removes the dynamic feature points of walking people, retaining only feature points of the static background. ORB-SLAM3 uses a feature point extraction method based on BRIEF descriptors, which can process large-scale scenes in real time but is easily affected by motion blur in dynamic scenes. LVID-SLAM combines semantic and geometric information and uses a dynamic feature point culling module to more accurately identify and cull the feature points of dynamic objects.

**Figure 10:**
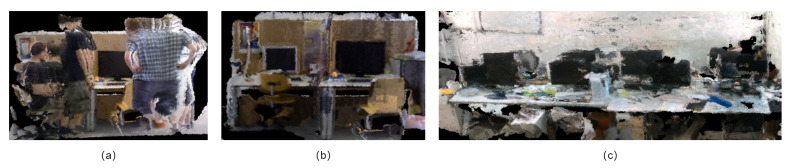
Octree dense mapping. (**a**) Dynamic objects are not considered for culling. (**b**) Dynamic objects are considered for culling. (**c**) A map built in our own dataset.

The mapping capability is primarily measured by the TUM RGB-D dataset. The dataset consists of two moving pedestrians walking around an office desk, with the camera continuously translating along the x, y, and z axes. Since the original ORB-SLAM3 could only construct sparse point cloud maps, the primary objective of testing on this dataset was to validate the construction of dense maps and the mapping performance after dynamic object filtering. Figure 10a shows a dense point cloud map without dynamic filtering, where human ghosting appears, significantly affecting mapping. In Figure 10b, dynamic object filtering has been applied, effectively removing the impact of moving people on mapping. The map includes most of the static objects in the scene. In Figure 10c, a map is created using our own dataset, and the map scene is mainly in the office.

**Figure 11:**
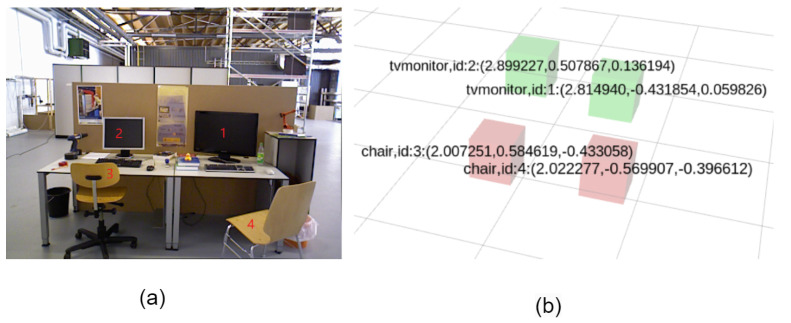
Semantic mapping. (**a**) Original reference image. (**b**) Semantic map.

Semantic mapping is performed while dense mapping is carried out, making use of the semantic information gathered from the object detection thread. The map contains object names, enabling mobile robots to recognize target objects, and location coordinate information, facilitating later path planning and navigation, enabling mobile robots to avoid obstacles and navigate to designated targets. In Figure 11a, the original image is shown, and the numbers correspond to the IDs assigned to objects by object detection in Figure 11b. In Figure 11b, green represents the display, red represents the chair, and similar objects can be accurately identified and assigned different IDs, while the position coordinates of the identified objects are also displayed.

### 4.5. Runtime Comparison

The LVID-SLAM design philosophy is primarily suited for systems requiring real-time operation. SLAM systems must strictly ensure operational efficiency without impairing the normal operation of the entire system, such as navigation, or affecting the system’s smooth operation. Therefore, we tested how much time it took the system to handle each frame on average and compared this with the time taken by other systems. As shown in Table 8, ORB-SLAM3, which does not include any semantic preprocessing modules, has the shortest processing time per frame. Semantic segmentation techniques at the pixel level, such as those employed by DS-SLAM and RDS-SLAM, enable more precise dynamic object segmentation. However, these systems incur higher frame processing time costs. The LVID-SLAM system, excluding ORB-SLAM3, achieves an average frame processing time of 23.2 ms, significantly improving frame processing efficiency while ensuring real-time operation.

## 5. Conclusions and Outlook

The LVID-SLAM method proposed in this paper is a novel, real-time semantic SLAM approach for dynamic scenes. Based on ORB-SLAM3, this method innovatively adds object detection and semantic mapping threads to form a multi-threaded, parallel processing mechanism. This ensures the system’s real-time capabilities when dealing with dynamic objects. It uses object detection to assist geometric constraints and more accurately filter out dynamic feature points. Concurrently, it leverages high-frequency IMU prior information to assist in correcting low-frequency image information, thereby resolving the issue of visual geometry degradation along polar lines, and further boosting the robustness of the system in high-dynamic scenarios. On the basis of sparse mapping, semantic dense octree mapping is added, which is conducive to the navigation of robots in the later stages. Experimental results show that LVID-SLAM, using RGB-D and stereo images as inputs, is superior to traditional algorithms in both outdoor and indoor comparison tests. Ablation experiments demonstrate that the combination of geometric modules and deep learning significantly improves system performance. LVID-SLAM demonstrates outstanding performance and broad applicability in SLAM tasks in dynamic environments. There are still some issues that we are investigating, such as insufficient generalization ability of object detection and the lack of instance segmentation for accurate segmentation. We plan to further investigate lidar in terms of sensors and deploy the algorithm on drones or unmanned vehicles.

## Figures and Tables

**Table 1:** Different empirical thresholds correspond to different pose errors.

ξ	0.05	0.1	0.2	0.3	0.4	0.5	0.6	0.7	0.8	0.9	1.0
Pose Error (m)	0.0183	0.0177	0.0181	0.0187	0.0173	0.0220	0.0251	0.1786	0.2136	0.3979	0.4250

**Table 2:** Results of ORB-SLAM3 and LVID-SLAM on ATE (M) on the TUM RGB-D dataset.

Sequences	ORB-SLAM3				LVID-SLAM				Improvement (%)			
	RMSE	Mean	Median	S.D.	RMSE	Mean	Median	S.D.	RMSE	Mean	Median	S.D.
fr3_walking_xyz	0.8090	0.6675	0.5740	0.4571	0.0164	0.0143	0.0131	0.0080	97.97	97.86	97.72	98.25
fr3_walking_static	0.1596	0.1426	0.1663	0.0718	0.0083	0.0071	0.0064	0.0043	94.81	95.05	96.15	93.95
fr3_walking_rpy	0.6726	0.5642	0.5506	0.3661	0.0882	0.0705	0.0505	0.0530	86.89	87.51	90.83	85.53
fr3_walking_half	0.2925	0.2395	0.1922	0.1679	0.0368	0.0307	0.0252	0.0202	87.43	87.18	86.89	87.95
fr3_sitting_static	0.0106	0.0095	0.0089	0.0048	0.0086	0.0075	0.0066	0.0043	18.54	20.79	26.47	10.40

**Table 3:** Results of RPE (°) rotation for ORB-SLAM3 and LVID-SLAM on TUM RGB-D dataset.

Sequences	ORB-SLAM3				LVID-SLAM				Improvement (%)			
	RMSE	Mean	Median	S.D.	RMSE	Mean	Median	S.D.	RMSE	Mean	Median	S.D.
fr3_walking_xyz	14.5988	12.9911	14.0647	6.6599	0.7199	0.6899	0.7183	0.2056	95.07	94.69	94.89	96.91
fr3_walking_static	3.4437	2.9956	2.3460	1.6988	0.3206	0.3205	0.3205	0.0054	90.69	89.30	86.34	99.68
fr3_walking_rpy	9.1173	7.4932	8.4483	5.1940	1.3865	1.0988	0.8776	0.8455	84.79	85.34	89.61	83.72
fr3_walking_half	6.2519	3.6047	1.1903	5.1081	1.3214	1.1836	1.0044	0.5874	78.86	67.16	15.62	88.50
fr3_sitting_static	0.6202	0.5008	0.5008	0.3659	0.4143	0.4027	0.4027	0.0975	33.20	19.59	19.59	73.36

**Table 4:** Results of RPE (M) translation for ORB-SLAM3 and LVID-SLAM on TUM RGB-D dataset.

Sequences	ORB-SLAM3				LVID-SLAM				Improvement (%)			
	RMSE	Mean	Median	S.D.	RMSE	Mean	Median	S.D.	RMSE	Mean	Median	S.D.
fr3_walking_xyz	0.5105	0.4231	0.3433	0.2856	0.0253	0.0221	0.0234	0.0123	95.05	94.78	93.19	95.70
fr3_walking_static	0.1660	0.1217	0.0819	0.1128	0.0123	0.0110	0.0086	0.0056	92.58	90.99	89.48	95.02
fr3_walking_rpy	0.4157	0.3337	0.3214	0.2478	0.0642	0.0556	0.0482	0.0321	84.55	83.34	85.01	87.03
fr3_walking_half	0.2871	0.1697	0.0583	0.2316	0.0455	0.0424	0.0399	0.0165	84.15	75.01	31.55	92.88
fr3_sitting_static	0.0176	0.0158	0.0158	0.0079	0.0116	0.0108	0.0091	0.0041	34.26	31.33	42.63	47.68

**Table 5:** Results of RPE (M) translation for ORB-SLAM3 and LVID-SLAM on TUM RGB-D dataset.

Sequences	ORB-SLAM3		DS-SLAM		RDS-SLAM		LVID-SLAM	
	RMSE	S.D.	RMSE	S.D.	RMSE	S.D.	RMSE	S.D.
fr3_walking_xyz	0.8090	0.4571	0.0247	0.0161	0.0571	0.0229	0.0164	0.0080
fr3_walking_static	0.1596	0.0718	0.0081	0.0036	0.0206	0.0120	0.0083	0.0043
fr3_walking_rpy	0.6726	0.3661	0.4442	0.2350	0.1604	0.0873	0.0882	0.0530
fr3_walking_half	0.2925	0.1679	0.0303	0.0159	0.0807	0.0454	0.0368	0.0202
fr3_sitting_static	0.0106	0.0048	0.0065	0.0033	0.0084	0.0043	0.0086	0.0043

**Table 6:** Comparison of the results of ATE (M ) on the KITTI dataset.

Sequences	ORB-SLAM3				LVID-SLAM				Improvement (%)			
	RMSE	Mean	Median	S.D.	RMSE	Mean	Median	S.D.	RMSE	Mean	Median	S.D.
00	7.59	6.83	6.62	3.31	4.06	3.68	3.78	1.71	46.47	46.04	42.91	48.36
01	35.64	27.98	21.66	22.08	8.78	8.27	8.00	2.93	75.37	70.42	63.04	86.73
02	9.12	7.75	7.04	4.81	6.70	6.34	6.70	2.16	26.55	18.12	4.80	55.22
03	2.93	2.48	2.03	1.56	2.65	2.31	2.12	1.29	9.61	6.90	−4.42	16.93
04	0.72	0.65	0.58	0.31	0.71	0.61	0.52	0.36	1.29	5.95	11.40	−16.87
05	1.68	1.56	1.52	0.62	1.80	1.65	1.48	0.73	−7.22	−5.44	2.22	−17.91
06	3.36	2.78	2.45	1.87	2.55	2.21	2.17	1.28	23.89	20.73	11.21	31.40
07	0.79	0.72	0.74	0.33	0.67	0.61	0.59	0.27	15.62	15.61	19.86	15.68
08	13.93	13.45	12.84	3.65	3.36	3.04	2.93	1.44	75.86	77.38	77.16	60.63
09	4.23	3.76	3.84	1.94	3.41	3.24	3.28	1.07	19.31	13.83	14.53	44.67
10	6.27	5.07	4.09	3.68	2.09	1.89	1.89	0.88	66.66	62.68	53.67	75.99

**Table 7:** Ablation experiments on the TUM RGB-D dataset.

Sequences	ORB-SLAM3	Geometry	Object	Object + Geometry
RMSE	0.8090	0.1481	0.0188	0.0164
Mean	0.6675	0.1462	0.0161	0.0143
Median	0.5740	0.1421	0.0141	0.0131
S.D.	0.4571	0.0236	0.0093	0.0080

**Table 8:** Results of runtime comparison.

Systems	Tracking Time Per Frame (ms)	Hardware Platform
ORB-SLAM3	13.2	AMD Ryzen 7 8745H, NVIDIA GeForce RTX 4060
DS-SLAM	76.5	Intel i7 CPU, P4000 GPU
RDS-SLAM	57.5	NVIDIA GeForce RTX 2080Ti
LVID-SLAM	23.2	AMD Ryzen 7 8745H, NVIDIA GeForce RTX 4060

## Data Availability

Publicly available datasets were analyzed in this study. The dataset can be found here: http://vision.in.tum.de/data/datasets/rgbd-dataset, https://www.cvlibs.net/datasets/kitti/ (all accessed on 1 May 2025).

## References

[1] Davison A.J., Reid I.D., Molton N.D., Stasse O. (2007). MonoSLAM: Real-Time Single Camera SLAM. IEEE Trans. Pattern Anal. Mach. Intell..

[2] Klein G., Murray D. Parallel Tracking and Mapping for Small AR Workspaces. Proceedings of the 2007 6th IEEE and ACM International Symposium on Mixed and Augmented Reality.

[3] Campos C., Elvira R., Rodríguez J.J.G., Montiel J.M., Tardós J.D. (2021). Orb-Slam3: An Accurate Open-Source Library for Visual, Visual–Inertial, and Multimap Slam. IEEE Trans. Robot..

[4] Newcombe R.A., Lovegrove S.J., Davison A.J. DTAM: Dense Tracking and Mapping in Real-Time. Proceedings of the 2011 International Conference on Computer Vision.

[5] Engel J., Koltun V., Cremers D. (2018). Direct Sparse Odometry. IEEE Trans. Pattern Anal. Mach. Intell..

[6] Mur-Artal R., Tardós J.D. (2017). Orb-Slam2: An Open-Source Slam System for Monocular, Stereo, and Rgb-d Cameras. IEEE Trans. Robot..

[7] Bescos B., Facil J.M., Civera J., Neira J. (2018). DynaSLAM: Tracking, Mapping, and Inpainting in Dynamic Scenes. IEEE Robot. Autom. Lett..

[8] He K., Gkioxari G., Dollar P., Girshick R. Mask R-CNN. Proceedings of the 2017 IEEE International Conference on Computer Vision (ICCV).

[9] Yu C., Liu Z., Liu X.J., Xie F., Yang Y., Wei Q., Fei Q. DS-SLAM: A Semantic Visual SLAM towards Dynamic Environments. Proceedings of the 2018 IEEE/RSJ International Conference on Intelligent Robots and Systems (IROS).

[10] Liu W., Anguelov D., Erhan D., Szegedy C., Reed S., Fu C.Y., Berg A.C., Leibe B., Matas J., Sebe N., Welling M. (2016). SSD: Single Shot MultiBox Detector. Proceedings of the Computer Vision—ECCV 2016.

[11] Zhang T., Zhang H., Li Y., Nakamura Y., Zhang L. (2020). Flowfusion: Dynamic dense rgb-d slam based on optical flow. Proceedings of the 2020 IEEE International Conference on Robotics and Automation (ICRA).

[12] Stückler J., Behnke S. Efficient Dense 3D Rigid-Body Motion Segmentation in RGB-D Video. Proceedings of the British Machine Vision Conference 2013.

[13] Kim D.H., Han S.B., Kim J.H., Kim J.H., Yang W., Jo J., Sincak P., Myung H. (2015). Visual Odometry Algorithm Using an RGB-D Sensor and IMU in a Highly Dynamic Environment. Robot Intelligence Technology and Applications 3.

[14] Yang X., Yuan Z., Zhu D., Chi C., Li K., Liao C. (2021). Robust and Efficient RGB-D SLAM in Dynamic Environments. IEEE Trans. Multimed..

[15] Hartley R., Zisserman A. (2003). Multiple View Geometry in Computer Vision.

[16] Tan W., Liu H., Dong Z., Zhang G., Bao H. (2013). Robust Monocular SLAM in Dynamic Environments. Proceedings of the 2013 IEEE International Symposium on Mixed and Augmented Reality (ISMAR).

[17] Fischler M.A., Bolles R.C. (1981). Random Sample Consensus: A Paradigm for Model Fitting with Applications to Image Analysis and Automated Cartography. Commun. ACM.

[18] Wang R., Wan W., Wang Y., Di K. (2019). A New RGB-D SLAM Method with Moving Object Detection for Dynamic Indoor Scenes. Remote Sens..

[19] Fan Y., Zhang Q., Tang Y., Liu S., Han H. (2022). Blitz-SLAM: A Semantic SLAM in Dynamic Environments. Pattern Recognit..

[20] Cui L., Ma C. (2019). SOF-SLAM: A Semantic Visual SLAM for Dynamic Environments. IEEE Access.

[21] Ji Q., Zhang Z., Chen Y., Zheng E. (2024). DRV-SLAM: An Adaptive Real-Time Semantic Visual SLAM Based on Instance Segmentation Toward Dynamic Environments. IEEE Access.

[22] Liu Y., Miura J. (2021). RDS-SLAM: Real-Time Dynamic SLAM Using Semantic Segmentation Methods. IEEE Access.

[23] Zhong F., Wang S., Zhang Z., Chen C., Wang Y. Detect-SLAM: Making Object Detection and SLAM Mutually Beneficial. Proceedings of the 2018 IEEE Winter Conference on Applications of Computer Vision (WACV).

[24] Wu W., Guo L., Gao H., You Z., Liu Y., Chen Z. (2022). YOLO-SLAM: A Semantic SLAM System towards Dynamic Environment with Geometric Constraint. Neural Comput. Appl..

[25] Farhadi A., Redmon J. (2018). Yolov3: An Incremental Improvement. Proceedings of the Computer Vision and Pattern Recognition.

[26] He J., Li M., Wang Y., Wang H. (2023). OVD-SLAM: An Online Visual SLAM for Dynamic Environments. IEEE Sens. J..

[27] Liu J., Li X., Liu Y., Chen H. (2022). RGB-D Inertial Odometry for a Resource-Restricted Robot in Dynamic Environments. IEEE Robot. Autom. Lett..

[28] Xiao L., Wang J., Qiu X., Rong Z., Zou X. (2019). Dynamic-SLAM: Semantic Monocular Visual Localization and Mapping Based on Deep Learning in Dynamic Environment. Robot. Auton. Syst..

[29] Zheng Z., Lin S., Yang C. (2024). RLD-SLAM: A Robust Lightweight VI-SLAM for Dynamic Environments Leveraging Semantics and Motion Information. IEEE Trans. Ind. Electron..

[30] Cheng S., Sun C., Zhang S., Zhang D. (2023). SG-SLAM: A Real-Time RGB-D Visual SLAM toward Dynamic Scenes with Semantic and Geometric Information. IEEE Trans. Instrum. Meas..

[31] Pan Z., Hou J., Yu L. (2023). Optimization RGB-D 3-D Reconstruction Algorithm Based on Dynamic SLAM. IEEE Trans. Instrum. Meas..

[32] Liu C., Chen X.F., Bo C.J., Wang D. (2022). Long-Term Visual Tracking: Review and Experimental Comparison. Mach. Intell. Res..

[33] Bescos B., Campos C., Tardós J.D., Neira J. (2021). DynaSLAM II: Tightly-coupled multi-object tracking and SLAM. IEEE Robot. Autom. Lett..

[34] Zhang J., Henein M., Mahony R., Ila V. (2020). VDO-SLAM: A visual dynamic object-aware SLAM system. arXiv.

[35] Hornung A., Wurm K.M., Bennewitz M., Stachniss C., Burgard W. (2013). OctoMap: An Efficient Probabilistic 3D Mapping Framework Based on Octrees. Auton. Robot..

[36] Everingham M., Van Gool L., Williams C.K.I., Winn J., Zisserman A. (2010). The Pascal Visual Object Classes (VOC) Challenge. Int. J. Comput. Vis..

[37] Forster C., Carlone L., Dellaert F., Scaramuzza D. (2017). On-Manifold Preintegration for Real-Time Visual–Inertial Odometry. IEEE Trans. Robot..

[38] A Benchmark for the Evaluation of RGB-D SLAM Systems. https://ieeexplore.ieee.org/document/6385773.

[39] Geiger A., Lenz P., Stiller C., Urtasun R. (2013). Vision Meets Robotics: The KITTI Dataset. Int. J. Robot. Res..

